# Brain Imaging of Taste Perception in Obesity: a Review

**DOI:** 10.1007/s13668-019-0269-y

**Published:** 2019-04-04

**Authors:** Christopher Kure Liu, Paule Valery Joseph, Dana E. Feldman, Danielle S. Kroll, Jamie A. Burns, Peter Manza, Nora D. Volkow, Gene-Jack Wang

**Affiliations:** 1 Laboratory of Neuroimaging, National Institute on Alcohol Abuse and Alcoholism, National Institutes of Health, 10 Center Dr, Rm B2L124, Bethesda, MD 20892-1013 USA; 2 Sensory Science and Metabolism Unit, Biobehavioral Branch, National Institute of Nursing Research, National Institutes of Health, 31 Center Drive, Rm 5B03, Bethesda, MD 20892-2178 USA; 3 National Institute on Drug Abuse, National Institutes of Health, 6001 Executive Blvd., Suite 5274, Bethesda, MD 20892-9581 USA

**Keywords:** Obesity, Neuroimaging, Taste, Gustation, Eating, Nutrition

## Abstract

**Purpose of Review:**

We summarize neuroimaging findings related to processing of taste (fat, salt, umami, bitter, and sour) in the brain and how they influence hedonic responses and eating behaviors and their role in obesity.

**Recent Findings:**

Neuroimaging studies in obese individuals have revealed alterations in reward/motivation, executive control/self-regulation, and limbic/affective circuits that are implicated in food and drug addiction. Psychophysical studies show that sensory properties of food ingredients may be associated with anthropometric and neurocognitive outcomes in obesity. However, few studies have examined the neural correlates of taste and processing of calories and nutrient content in obesity.

**Summary:**

The literature of neural correlated of bitter, sour, and salty tastes remains sparse in obesity. Most published studies have focused on sweet, followed by fat and umami taste. Studies on calorie processing and its conditioning by preceding taste sensations have started to delineate a dynamic pattern of brain activation associated with appetition. Our expanded understanding of taste processing in the brain from neuroimaging studies is poised to reveal novel prevention and treatment targets to help address overeating and obesity.

## Introduction

The prevalence of obesity in the United States has risen significantly since the 1980s [1]. Between 2015 and 2016, 4 in 10 Americans were considered obese, which is defined in the USA as having a body mass index (BMI) over 30, and over 28 in China [2••, 3]. Obesity-related diseases are some of the leading causes of preventable death in the USA and world, so it is paramount to understand their underlying causes [4, 5]. With the increasing presence of refined sugars, salts, and fats and the high calorie content in processed foods, individual, neurobiological differences in taste perception and conditioning to these ingredients may contribute to increased risk for obesity [6, 7, 8•, 9–11]. To that end, neuroimaging and psychophysical studies could reveal neural mechanisms of aberrant eating in obesity.

Sensory systems, particularly taste and smell, significantly affect food selection and consumption. Taste sensation occurs when chemosensory stimuli (e.g., salt) interact with taste receptor cells (TRCs) in the tongue. In addition to the five basic tastes (sweet, salty, bitter, sour, and umami), which are chemotopically organized in the human gustatory cortex, fat is considered a sixth taste modality [12–16]. Taste information is sent to the brain’s feeding and reward systems, which affect eating behaviors and taste preferences [17, 18]. Hedonic eating, or food consumption for pleasure but not hunger, when excessive, can lead to weight gain and obesity disrupting processing of taste input to the brain and enhancing conditioning to taste, smell, and calorie processing [11, 19–23]. When it comes to smell, olfactory ablation protects against diet-induced obesity in preclinical studies, suggesting that smell modulates taste perception to influence food intake [24]. Additionally, alterations in reward, executive control and affective circuits, and in homeostatic signals are risk factors for excessive hedonic eating [25]. Aberrant dopamine signaling akin to that observed in addiction is associated with these disruptions, which may further contribute to compensatory reward-seeking behaviors like overeating [26–28].

**Fig. 1:**
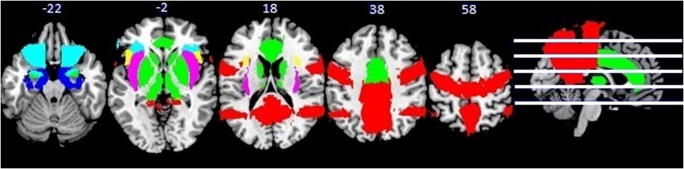
Brain activation patterns (overweight and obese > healthy controls) of taste modalities (bitter, salty, and fat) compiled from studies in Table 1. **Red** (*fat taste*): BOLD contrast (overweight > healthy controls) to high-fat, high-sugar milkshake in right insula/operculum, precentral gyrus, angular gyrus, bilateral precuneus, posterior cingulate (Bohon et al., 2017). **Green** (*bitter taste*): BOLD contrast (obese > healthy controls) to quinine-hydrochloride solution in insula, ACC, OFC, amygdala, putamen, pallidum (Szalay et al. 2012). **Blue** (*salty taste*): [^18^F-FDG] glucose metabolism (overweight and obese > healthy controls) following sodium chloride solution in insula, OFC, parahippocampus (Li et al., 2017). **Pink**: Overlapping brain activation in insula in response to fat, salty, and bitter tastes. **Turquoise**: Overlapping brain activation in OFC in response to bitter and salty tastes. **Yellow**: Overlapping brain activation patterns in response to fat and bitter tastes the numbers above the brain slices indicate the Z-coordinate in MNI space

Psychophysical methods (e.g., whole mouth sip and spit of tastants) have been extensively used to study taste. However, advances in neuroimaging procedures and chemosensory stimulus delivery techniques (e.g., tastant delivery during brain imaging sessions) have provided novel insight into central mechanisms underlying taste and hedonic eating. Brain responses to food stimuli may differ in obese individuals compared to those without obesity, as illustrated in Fig. 1. Additionally, individuals with obesity show marked structural and functional brain-circuitry alterations [29–36].

Here, we review the impact of taste on neuroimaging outcomes regarding the hedonic aspects of eating in obesity, with a focus on the neural correlates of fat, umami, bitter, salty, and sour tastes across brain areas implicated in obesity, as outlined in Table 1. Although sweet is one of the basic tastes, the neural correlates of hedonic responses to sugar in obesity were recently reviewed so we do not discuss them here [37, 38]. Further, evidence of compromised olfaction capacity in obesity exists [39, 40]. However, few studies have assessed brain activation patterns to smell in individuals with obesity [41–43]. The brain regions we focus on include the primary and secondary gustatory cortices. The primary gustatory cortex processes taste perception and comprises the anterior insula and frontal operculum. The secondary gustatory cortex, which comprises the orbitofrontal cortex (OFC) and medial prefrontal cortex (mPFC), integrates sensory properties of food to generate flavor and assigns saliency values [44].

**Table 1:** Neural correlates of taste information processing in obese and overweight populations

Authors (year)	Study group	Imaging task	Neural correlates in obese and overweight populations vs healthy controls
Fat taste			
Bohon et al. (2017)	*N* = 10 healthy-weight children (BMI between 5th and 85th percentile) *N* = 8 overweight children (BMI greater than 85th percentile)	Visual food cue presentation of either chocolate milkshake or water Tastant delivery of either chocolate milkshake or tasteless solution (3 T fMRI)	Greater BOLD contrast (milkshake > tasteless) in right insula, operculum, precentral gyrus, angular gyrus, bilateral precuneus, and posterior cingulate
Babbs et al. (2013)	*N* = 12 normal-weight adults (BMI < 25) *N* = 13 overweight adults (BMI ≥ 25)	Tastant delivery of either milkshake or tasteless solution Self-report measures of impulsivity, willingness to work for food, and pleasantness (rated during scan) of milkshake taste (3 T fMRI)	Greater BOLD contrast (milkshake > tasteless) in ventral putamen Lower BOLD contrast (milkshake > tasteless) in caudate nucleus
Umami taste			
Magerowski et al. (2018)	*N* = 30 healthy-weight women (mean BMI: 22.1 ± 0.4) *N* = 30 Three Factor Eating Questionnaire at baseline Randomization into buffet meal test or fMRI paradigm after MSG+/MSG- broth consumption *N* = 15 buffet meal tests *N* = 14 fMRI paradigm	Visual food cue presentation that subjects rated on health and appetitiveness. Choice selection followed visual food cue presentation where subjects presented with two images of food and told to choose the food they would “rather eat right now” (3 T fMRI)	Greater BOLD contrast (MSG+ > MSG-) in left DLPFC and lower BOLD contrast (MSG+ > MSG-) in cerebellum, precuneus, and fusiform gyrus in women with high eating disinhibition *note: all subjects are healthy volunteers
Salty taste			
Li et al. (2017)	*N* = 156 healthy-weight participants *N* = 100 overweight participants (BMI ≥ 25) and participants with obesity (BMI ≥ 28)	Buccal administration of sodium chloride stimulus solution 40 min prior to scan ([^18^F-FDG]-PET/CT)	Greater glucose metabolism in insula, OFC, and parahippocampus
Hardikar et al. (2018)	*N* = 30 healthy-weight participants (BMI between 18.5 and 25) *N* = 25 participants with obesity (BMI > 30)	Tastant delivery of suprathreshold sucrose or sodium chloride solution (EEG)	Weaker and shorter-latency gustatory-evoked potential to sodium chloride
Bitter taste			
Szalay et al. (2012)	*N* = 12 healthy-weight participants (mean BMI 21.42 ± 2.53) *N* = 12 participants with obesity (mean BMI: 34.05 ± 3.35)	Tastant delivery of either quinine hydrochloride or tasteless distilled water (3 T fMRI)	Greater brain activation in ACC, gustatory cortex, OFC, amygdala, putamen, and pallidum
Green et al. (2015)	*N* = 15 healthy-weight participants (mean BMI: 25.25 ± 3.3) *N* = 16 individuals with abdominal obesity (mean BMI: 39.26 ± 2.2)	Tastant delivery of either caffeine or sucrose solutions (3 T fMRI)	Greater brain activation in pre- and post- central gyri, fusiform gyrus, insula, lentiform nucleus, putamen, and frontopolar cortex to caffeine and sucrose
Sour taste			
N/A	N/A	N/A	N/A

## Fat Taste

Palatable, high-fat foods contribute to increased risk for obesity [45]. Recently, orosensory fat perception was proposed as a primary taste that might regulate dietary fat consumption [13–16]. Although several taste receptors for fat exist, CD36, a scavenger receptor with an affinity for long chain fatty acids, is the most studied [13]. CD36 is expressed on the membrane of lingual TRCs and facilitates fatty acid uptake [46]. Preclinical *CD36* knockout studies indicate that the receptor is necessary to establish fatty taste preferences [47]. Additionally, *CD36* genetic variations have been associated with obesity in humans [13].

Recent neuroimaging studies in healthy volunteers investigated the brain’s response to fatty stimuli. Several studies found that oral fat administration was associated with blood-oxygen-level dependent (BOLD) activation in the secondary gustatory cortex [48, 49]. Support from source-localized electroencephalography (EEG) studies shows that consumption of high-fat relative to low-fat milk was associated with greater late positive potential amplitudes in OFC and hippocampus, which reflected calorie content assessment of milk [50]. However, compared to sugar, fat may affect brain reward regions differently. For example, high fat relative to high-sugar milkshakes elicited greater BOLD responses in caudate and somatosensory regions, but no significant bilateral insular changes, whereas high sugar relative to high-fat milkshakes elicited greater BOLD responses in putamen and gustatory regions and increased bilateral insula activation [51]. Based on these findings, it appears that fat might affect brain reward circuitry differently than sugar.

Further, obesity is associated with reward circuitry dysregulations, which is reflected in brain response to fat [30, 52, 53]. For instance, overweight compared to healthy-weight children showed greater BOLD responses in right insula, operculum, bilateral precuneus, and posterior cingulate cortex following milkshake consumption [9]. Additionally, following milkshake consumption, overweight compared to healthy-weight adults showed greater BOLD responses in ventral putamen and rolandic operculum but lower caudate BOLD responses, which were associated with higher impulsivity [10]. This suggests a mechanism for compulsive eating [10]. However, these milkshakes had high sugar and fat content, making it unclear whether these brain response patterns reflect an interaction between the tastants [9, 10].

Brain responses to fat in healthy volunteers can also be used to elucidate eating patterns and BMI gain. Eldeghaidy et al. [54] showed that amygdala BOLD response to a fatty stimulus is attenuated after a high-fat meal compared to water intake. This suggests that satiety from a high-fat meal reduces the reward response to fat. Moreover, reduced BOLD response to a high-fat, low-sugar milkshake relative to a tasteless solution in the pre-supplementary motor area, a region critical for inhibitory control, predicted BMI gain in healthy volunteers [55]. Similar studies should be replicated in individuals with obesity in order to understand how these processes differ between individuals with and without obesity.

## Salty Taste

Excess dietary salt has been linked to obesity and its comorbidities [56–58]. Altered salt perception may be a risk factor for, or a result, of obesity, as altered salt taste perception has been reported in obese individuals by some investigators but not by others [58–62]. Salt taste is generated by the salt-sensitive epithelial sodium channel (ENaC) and transient receptor potential cation channel subfamily V member 1 (TRPV1) [63–65]. While ENaC regulates appetitive responses to low salt concentrations, TRPV1 regulates aversive responses to high salt concentrations in part via bitter and sour TRCs [66–68]. This concentration-dependent response helps maintain optimal sodium balance and coincides with a negative association between salt taste intensity and hedonic ratings [69–71].

Functional magnetic resonance imaging (fMRI) studies demonstrate that salty tastes engage various brain regions, including the frontal operculum, amygdala, OFC, middle cingulate cortex, thalamus, pre- and postcentral gyrus, and dorsolateral prefrontal cortex (DLPFC) [72, 73]. These regions modulate reward, taste processing, and executive control in eating. For example, increased activity in the left DLPFC, a region implicated in executive function, has been associated with improved self-control during food choice selection [74–77]. These regions also encode salt taste intensity: [H_2_^15^O]-PET (positron-emission tomography), which measures cerebral blood flow, and fMRI studies reveal that sodium chloride in both highly concentrated, aversive levels and low, non-aversive levels engages the amygdala and OFC [72, 78]. For example, the middle insula and amygdala are increasingly engaged as sodium concentrations increase and hedonic ratings decrease [79]. Thus, a range of salt intensities recruit similar circuitry despite varied subjective responses. Additionally, salt taste intensity modulates the strength of insular-thalamic effective connectivity and is negatively associated with hedonic ratings, suggesting a mechanism for aversion to high salt intensities [68, 69].

Whether salt taste perception is different in obesity remains uncertain due to mixed psychophysical findings. Several studies showed no significant salt taste sensitivity differences between individuals with and without obesity [61, 62]. In studies that report differences, some studies show higher salt sensitivity in obese individuals compared to those without obesity [80, 81]. Yet, other studies report lower salt sensitivity in obese individuals compared to those without obesity [8•, 82, 83]. Additionally, studies on salt taste preferences have yielded mixed results in individuals with obesity [8•, 80, 81]. These discrepancies may be due to methodological differences. Specifically, studies that found lower salt sensitivity in individuals with obesity used taste strips or food stimuli; the textual properties of these stimuli may have confounded taste responses [8•, 82, 83]. Furthermore, in studies that report higher or similar salt sensitivities in obese compared to non-obese individuals, stimulus delivery patterns may have also confounded results; whereas some studies had participants rinse the oral cavity with aqueous solutions of sodium chloride, other studies had solutions sipped or directly administered to the tongue [62, 80, 81].

To date, studies on neural responses to salty taste in obesity have been limited. In an [^18^F-Fluorodeoxyglucose (FDG)]-PET study to measure brain glucose metabolism (marker of brain function), patients with obesity showed greater OFC, insular, and parahippocampal metabolism in response to a salty taste stimulus compared to healthy controls [8•]. Increased activity in these regions was associated with reduced salt sensitivity, higher salt preference, and greater salt intake in patients with obesity [8•]. Although no perceptual differences were reported, an EEG study revealed that obese compared to non-obese individuals showed weaker and shorter-latency gustatory-evoked potentials in response to salty taste [84]. Thus, salt consumption in obesity may be associated with alterations in reward, gustatory, and memory-related areas.

## Umami Taste

Umami contributes to a sense of satiety [85]. Due to its role in signaling satiety, umami has been examined in the context of weight-loss interventions and obesity [86]. Umami is a savory flavor transmitted by monosodium L-glutamate (MSG), and is recognized as one of five basic tastes largely through glutamate-binding G protein-coupled receptors and amino acid and nucleoside sensing receptors in taste buds [87–89]. Umami receptors are also expressed in the gut such that glutamate consumption is related via a vague-nerve mediated signaling pathway to the forebrain, where feeding behaviors are regulated [86, 90–94].

Studies show that umami contributes to satiety, as consumption of MSG-added (MSG+) broth increased subjective satiety in healthy-weight women compared to MSG-absent (MSG-) broth [95]. Whether this affects subsequent caloric intake is mixed. Across women with and without obesity, MSG+ soup was associated with lower calorie consumption at mealtime relative to MSG- soup [96, 97]. Women with high eating disinhibition, as assessed by the Three Factor Eating Questionnaire, consumed less saturated fat in a buffet meal following consumption of MSG+ relative to MSG- broth [86]. However, some studies report no effect of MSG on subsequent calorie intake [86, 95]. Together, these findings suggest that MSG influences satiety and possibly calorie consumption, especially in individuals with obesity or individuals at risk of weight gain [86, 96].

Some studies suggest that individuals with obesity have reduced sensitivity but higher preference for umami taste compared to individuals without obesity. Women and adolescents with obesity required higher MSG concentrations for umami taste detection compared to healthy controls [83, 98]. However, another study found that umami taste detection did not vary by BMI [22]. Interestingly, Van Langeveld et al. [99] found that individuals with obesity obtain a larger percentage of daily calories from “salt, umami, and fat”-tasting foods and less from “sweet and fat”-tasting foods than individuals without obesity reflecting perhaps a higher preference for umami-tasting foods in obesity.

fMRI studies in healthy volunteers have demonstrated the effects of umami taste on brain activation patterns. Umami and glucose taste stimuli result in similar BOLD activation patterns, including the insular/opercular cortex and the caudolateral OFC, but umami resulted in greater BOLD activation in the rostral anterior cingulate cortex (ACC), a region involved in complex cognition and behavioral adjustment [100]. Increasing MSG concentration with an umami-enhancer, disodium 5′-inosinate, was associated with middle insula BOLD activation [101]. Other studies found both MSG and NaCl administration were associated with activation in the insula, operculum, pre- and postcentral gyrus, thalamus, supplementary motor area, and OFC [22, 102]. Moreover, umami and salty tastes were associated with stronger activation in the primary gustatory cortex in umami high-tasters compared to low-tasters [22]. Together, these findings suggest that umami and salty taste perception share a common processing system, and may thus have similar contributions to behaviors implicated in obesity.

As demonstrated with fMRI, relay of umami-related taste information to cortical areas involved in inhibitory control can further mediate subsequent food intake. In subjects with high eating disinhibition, consumption of MSG+ relative to MSG- broth resulted in increased BOLD response in the left DLPFC, but decreased BOLD responses in regions (cerebellum, precuneus, and fusiform gyrus) that have been associated with increased motivation and attention to food; these responses were associated with lower saturated fat intake in a subsequent buffet meal [86]. Additionally, cognitive functions influence umami taste perception and its representation in the brain. MSG exposure was associated with greater insula BOLD response when participants were instructed to remember and rate the intensity relative to the pleasantness of taste, but greater medial OFC and pregenual cingulate BOLD response when participants remembered and rated the pleasantness relative to the intensity of taste; thus, selective attention, may influence umami taste perception and its valuation in the brain [103].

## Bitter Taste

Bitter taste detection ability has been shown to influence dietary fat consumption, suggesting its potential relevance in obesity [104]. Bitter tastes are transduced by specialized G protein-coupled bitter taste receptors (T2Rs) [105–107]. From an evolutionary perspective, bitter tastes signal the presence of toxic food ingredients [70].

Bitter tastes affect various brain regions associated with appetite reduction. [H_2_^15^O]-PET, fMRI, and functional near-infrared spectroscopy studies have demonstrated that bitter tastes are associated with stronger recruitment of amygdala, OFC, DLPFC, frontal operculum, and bilateral ventrolateral PFC compared to tasteless solutions in healthy volunteers [108–110]. Likewise, DLPFC responses to bitter taste stimuli are associated with appetite reduction [108]. EEG studies have further shown that bitter relative to neutral tastes also reduce appetitive ratings to high-caloric food images and are reflected in blunted event-related potentials, including fronto-central late positive potentials [111, 112].

Conditioning to bitter tastes also modulates hedonic evaluation. For instance, the quantity of coffee intake per week was positively associated with right caudate BOLD response to caffeine, suggesting that habitual consumption of bitter-tasting substances recruits reward-related areas [113]. Interestingly, lower left ACC, right precuneus, and left superior frontal gyrus BOLD responses to caffeine in caffeine consumers compared to non-caffeine consumers were associated with lower ratings of bitter taste intensity [114]. These findings may be attributed to caffeine-induced modulation of adenosine receptor density in the caudate rather than the bitter taste [115]. Furthermore, presentation of a visual cue conditioned to be mildly aversive was associated with lower insula and operculum BOLD responses and predicted lower aversion ratings to a highly aversive, bitter-tasting quinine solution [116]. Thus, altering expectations can affect subsequent taste perception. Further, variations in bitter taste receptor genes may also affect bitter taste perception [117].

Studies in individuals with obesity show that brain activation patterns associated with bitter tastes may influence bitter taste hedonics. For example, individuals with obesity showed stronger ACC, insula and operculum, OFC, amygdala, putamen, and pallidum activation in response to quinine-hydrochloride compared to individuals without obesity [118]. Activation in these areas, which modulate reward, executive function, and gustatory processing, negatively correlated with hedonic scores, suggesting that quinine-hydrochloride downregulates hedonic responses to bitter tastes more so in individuals with obesity [118]. Compared to healthy controls, individuals with abdominal obesity also showed greater activation in sensory and higher-level taste processing regions while evaluating bitter taste hedonics; however, no differences in hedonic ratings were reported [119•]. Mixed findings on the interaction between brain responses and hedonic ratings to bitter tastes highlight the need for more consistent and reliable measures of taste preferences in human studies [120, 121].

## Sour Taste

Although less explored in the context of obesity, sour taste may play a role in food selection and consumption. Sour tastes are mostly registered by the polycystic-kidney disease-like ion channel, a proton channel that is sensitive to low-pH stimuli [122]. Stimulation of TRCs that contain this channel may mediate aversion to highly acidic concentrations to prevent further sour food ingestion [123, 124].

In healthy populations, fMRI studies show that sour taste stimuli, such as citric acid, recruit brain regions in an age-, sex-, and condition-dependent manner [125, 126]. For example, citric acid exposure was associated with increased caudate activation across all sated participants and in hungry females, but not in hungry males [125]. Further, neural responses to sour tastes differed by age, whereas hedonic ratings for sour tastes were independent of sex or condition [125]. Similarly, citric acid was associated with greater posterior insula BOLD response relative to water in younger compared to older adults [126].

While the neural correlates of sour taste in obesity are limited, psychophysical measures have provided insight. Adolescents with and without obesity displayed similar sensitivities to and recognition for citric acid [80, 83]. In contrast, adults with a BMI over 28 had significantly worse sour taste detection ability compared to adults with a BMI under 28 [62]. Given that sour tastes are programmed to signal toxic substances, these findings indicate the presence of long-term effects of obesity on sour taste processing and associated health outcomes [127].

## Clinical Implications and Research Opportunities

While chemosensory stimuli interact with TRCs in the tongue to generate taste perception in the brain, taste also results from the interaction of chemosensory stimuli with extraoral TRCs, including those in the gastrointestinal (GI) tract [128–130]. Although their function continues to be explored, GI taste receptors (i.e., sweet, bitter, fat, and amino acid receptors) appear to modulate satiety hormones [131–135]. Furthermore, dysregulation of chemosensory pathways in the gut may contribute to increased risk for obesity [136]. Given the importance of taste perception in neuroendocrine functions, TRCs in the GI tract could offer potential therapeutic targets in obesity [137••].

Intragastric administration of quinine and/or MSG can alter brain activation patterns in regions (insula, subcortical limbic and memory structures, homeostatic and hedonic centers) that regulate food intake [129, 130]. Further, intragastric administration of quinine can lead to lower hunger scores and hedonic eating, which were associated with changes in orexigenic and satiety gut hormone levels [128, 138, 139]. That gut taste receptor stimulation affects brain activation patterns underscores the importance of oral-nasal-brain-gut axis signaling in chemosensory pathways associated with appetite regulation, which may elucidate how nutritional interventions target these pathways in obesity.

Studies of bariatric surgery on taste activation patterns in obese populations lend further support for gut-brain axis signaling in postoral nutrient sensing, or appetition. Indeed, bariatric surgery has been shown to recover structural abnormalities and mu-opioid receptor density in the insula [140, 141]. Additionally, following bariatric surgery, the insula showed decreased resting-state activity compared to pre-bariatric surgery, which may reflect reduced interoceptive attention to hunger signals [142, 143].

While these studies show that vagal and gut-brain hormonal signaling promotes satiety to control food intake in a negative-feedback manner, appetition, a distinct process from satiation, can drive positive reinforcement of food intake [144••]. Conditioning studies in rodents have demonstrated that flavored solutions paired with intragastric infusion of palatable foods modulate flavor preferences, suggesting that gut-brain signaling mechanisms drive appetition and increased food intake via postoral nutrient-conditioned preferences [145–147]. More research on these mechanisms may help identify better targets for pharmacological blockade and subsequent reversal of nutrient-conditioned flavor preferences [144••].

Collectively, more research addressing the clinical implications of taste perception and extraoral chemosensory receptors in obesity are needed to understand how different interventions such as exercise may affect taste perception. For example, exercise has recently been associated with increased sensitivity and lower hedonic ratings to sweet and umami tastes [148••]. In adolescents with obesity, high intensity interval exercise was associated with lower fat and sweet taste preferences and lower fat implicit wanting, all of which contributed to reduced food intake [149]. With neuroimaging, the effects of exercise on taste and smell perception would help elucidate the neurobiological basis of exercise in the prevention of obesity. Such knowledge could potentially help clinicians use activity levels to guide personalized nutritional and dietary interventions for obesity in the future.

## Summary and Conclusion

In this review, we summarize the neural correlates of umami, salty, fat, bitter, and sour tastes that may be altered in obesity, as outlined in Table 1. While sweet taste is widely linked to obesity, additional primary tastes, particularly fat and umami, may also be implicated. Alterations in the neural correlates of the basic tastes, notably fat and umami, may reflect different hedonic responses to taste information in obesity. However, limited and conflicting findings of neuroimaging outcomes on bitter, salty, and sour tastes highlight their inconclusive role in obesity.

Fat tastes recruit reward, executive control, and gustatory brain regions that regulate dietary restraint [48–51]. Reduced neural responses to fat in these regions have been associated with increased intake of highly palatable food. Several fMRI studies have supported these findings, demonstrating differential BOLD responses to fat taste in participants who were obese or overweight compared to healthy controls, particularly in the gustatory cortex and reward pathway [9, 10]. Thus, hedonic eating in obesity may be driven by individual differences in the neural mechanisms of fat taste perception. Further, variations in the *CD36* taste receptor gene have been associated with obesity in humans [13].

Similar to fat, umami tastes recruit reward, executive control, gustatory, and salience networks in the brain, and are associated with satiety levels. MSG has been studied for its role in appetite suppression and calorie restriction [96, 97]. Further, fMRI studies suggest that MSG influences BOLD activation patterns in areas related to dietary restriction and attention to food [86]. Together, these findings highlight the relevance of umami in obesity in the context of weight loss. Studies of both fat and umami found links between taste stimuli-induced BOLD response and impulsivity, which further implicates taste perception in disinhibited eating and obesity [10, 86].

Although not discussed in this review, sweet tastes are associated with altered activation in reward, executive control, homeostatic, and affective brain regions in individuals with obesity. Obese compared to healthy-weight individuals show decreased perfusion in the PFC but increased perfusion in the hypothalamus following glucose and fructose consumption [150]. In addition, obese and overweight compared to healthy-weight individuals show differential BOLD response to sweet taste in reward, gustatory, and affective brain regions; however, whether sweet taste coincided with differences in hedonic and behavioral measures was mixed [9, 10, 119•, 151].

Together, these findings illustrate the neurobiological underpinnings of taste modalities and hedonics to food tastes and their relevance in obesity. While neuroimaging studies of fat and umami tastes show the most robust association with obesity in the present review, further studies on brain activation responses to sour, salty, and bitter tastes are needed to understand the interplay of primary taste perception in cognitive control over eating behaviors and dietary restraint. Given that smell modulates taste perception to influence food intake, more neuroimaging studies of smell in obesity are also needed. Indeed, application of odorant stimuli delivery techniques to neuroimaging procedures such as fMRI and EEG has started to gain traction in recent years [152, 153]. Further neuroimaging research is also needed to better characterize the influence that taste receptors in the gut have on brain responses following food consumption. A greater understanding of the brain responses to taste and smell in obesity may help inform prevention and treatment efforts.
